# ELISA‐based detection of immunoglobulins against extracellular vesicles in blood plasma

**DOI:** 10.1002/jex2.129

**Published:** 2024-03-07

**Authors:** Tom A. P. Driedonks, Linglei Jiang, Olesia Gololobova, Zhaohao Liao, Kenneth W. Witwer

**Affiliations:** ^1^ Department of Comparative and Molecular Pathobiology Johns Hopkins School of Medicine Baltimore Maryland USA

**Keywords:** ectosomes, ELISA, exosomes, extracellular vesicles, IgG, immunogenicity, microvesicles, plasma

## Abstract

Extracellular vesicles (EVs) are intensively investigated for their therapeutic potential and application as drug delivery vehicle. A broad perception of favourable safety profiles and low immunogenicity make EVs an attractive alternative to synthetic nanoparticles. We recently showed that repeated intravenous administration of human cell‐derived EVs into pig‐tailed macaques unexpectedly elicited antibody responses after three or more injections. This coincided with decreasing EV circulation time, and may thus hamper successful EV‐mediated cargo delivery into tissues. Here, we share the custom ELISA protocol that we used to measure such antibody responses. This protocol may help other researchers evaluate immune responses to EV‐based therapies in preclinical studies.

## 1 INTRODUCTION

Extracellular vesicles (EVs) are membrane‐enclosed, nano‐sized particles released by virtually all cells (Colombo et al., 2014). EVs are intensively investigated in part because of their intrinsic therapeutic potential (Lener et al., 2015; Witwer & Wolfram, 2021). Furthermore, EVs can be engineered to deliver therapeutic cargo, such as drug compounds, (si)RNA, CRISPR/Cas9, and viral vectors, into cells (György et al., 2014; Kim et al., 2017; O'Loughlin et al., 2017). It is generally recognized that EVs have a favourable safety profile (Jiang et al., 2022; Saleh et al., 2019; Zhu et al., 2017). It is also presumed that autologous EVs do not elicit immune responses (Elsharkasy et al., 2020; Saleh et al., 2019; Walker et al., 2019), whereas allogenic EVs, which display foreign antigens, may invoke immune responses (Bliss et al., 2020; Emerson et al., 2022; Jiang et al., 2022; Montaner‐Tarbes et al., 2018). In contrast, synthetic nanoparticles may elicit inflammatory reactions (reviewed by Irvine et al., 2015). For example, it was shown that polyethylene glycol (PEG) in liposomes elicited IgM antibody responses after repeated administration, resulting in accelerated blood clearance (ABC) of these particles (Dams et al., 2000; Ishida et al., 2006).

We recently showed that the circulation time of Expi293F‐derived EVs unexpectedly decreased after three or more intravenous administrations in pig‐tailed macaques (*Macaca nemestrina*) (Driedonks et al., 2022). The circulation time negatively correlated with plasma levels of EV‐specific IgG and IgM immunoglobulins, suggesting a mechanism similar to ABC (Driedonks et al., 2022). It remains unclear whether this phenomenon was caused by species differences between EV donor and recipient animal model, and whether this can be generalized to other experimental systems and cell lines. However, considering the large number of preclinical studies on human cell‐derived EVs in mice (Escudé Martinez de Castilla et al., 2021), it is important to investigate further whether repeated administration of EVs generally provokes antibody responses, and whether allogenic and autologous EVs differ in immune response.

Here, we present a custom ELISA protocol that we developed to measure anti‐EV immunoglobulins in macaque plasma, enabling researchers to measure anti‐EV immunoglobulins in plasma samples obtained during their in vivo experiments. The protocol can be adapted for other species and biological sample types. Using this protocol will help researchers to evaluate potential immune responses to EV‐based therapies and, where needed, to pursue strategies to circumvent potential ABC effects.

## 2 MATERIALS

- RIPA buffer: 25 mM Tris‐HCl pH 7.6 + 150 mM NaCl + 1% NP‐40 + 1% sodium deoxycholate + 0.1% SDS, (Thermo Fisher, cat# 89900)
- Complete mini EDTA‐free protease inhibitor tablets (Roche, cat# 11836170001)
- SuperBlock T20 blocking buffer (Thermo Fisher, cat# 37536)
- Wash buffer: 1× PBS + 0.05% Tween‐20 (Sigma‐Aldrich, cat# P7948) and 0.1% chloroacetamide (Sigma‐Aldrich, cat# C0267), pH 7.0. For the current protocol, we used wash buffer from the Perkin Elmer p24 ELISA kit (NEK050001KT) with the same composition.
- Assay Buffer (Thermo Fisher, cat# DS98200)
- 96‐well Clear Flat Bottom Polystyrene High Bind Microplates (Corning Cat # 9018)
- Adhesive ELISA plate covers
- anti‐monkey‐IgG‐HRP (LifeSpan Biosciences LS‐C56745)
- anti‐monkey‐IgM‐HRP (LifeSpan Biosciences LS‐C61207)
- 1‐Step Ultra‐TMB‐ELISA solution (Thermo Fisher cat# 34028); other TMB substrates could also be used.
- Stopping solution: 0.5N H2SO4
- Plate reader capable of measuring absorbance at 450 nm (for current protocol we used a BioRad iMark microplate reader (cat# 1681135))
- Benchtop centrifuge (for current protocol we used an Eppendorf 5415C centrifuge)

## 3 COLLECTION OF BLOOD AND PLASMA PROCESSING

1. Draw 500 µL blood from animal into 1.5 mL Eppendorf tube containing citrate‐dextrose solution (ACD, Sigma‐Aldrich, cat# C3821).
2. Gently mix by inverting five times.
3. Centrifuge 5 min at 800 g at room temperature in benchtop centrifuge.
4. Carefully collect the top‐layered plasma, store directly at −80°C until analysis.

## 4 ELISA PROTOCOL

Volumes given in the following protocol are sufficient for coating a full 96‐well plate with EVs. ELISA plates can be coated with EV lysates or intact EVs, depending on the research question. Both protocols are described below. For smaller/larger experiments, these volumes can be scaled down/up accordingly.

### 4.1 Day 1: Coat plate with EV lysates

1. Add 0.5 mL EV suspension (particle concentration of 1.8E10 EVs/mL) to 0.5 mL RIPA + protease inhibitors (one protease inhibitor tablet per 10 mL RIPA buffer).
2. Incubate EV lysate on ice for 30 min.
3. Dilute EV lysate with or PBS (or 1× Assay Buffer) to 10 mL.
4. Add 100 µL diluted EV lysate per well (equivalent of 1.8E8 EVs/well) to coat the plate.
5. Close the plate using an adhesive seal.
6. Incubate overnight at 4°C to coat the plate.

### 4.2 Day 1: Alternatively: Coat plate with intact EVs

1.1 Add 0.5 mL EV suspension (recommended particle concentration of 1.8E10 EVs/mL) to 9.5 mL PBS.
1.2 Add 100 µL diluted EVs per well (equivalent of 1.8E8 EVs/well) to coat the plate.
1.3 Close the plate using an adhesive seal.
1.4 Incubate overnight at 4°C to coat the plate.

### 4.3 Day 2: ELISA‐based detection of immunoglobulins

7. Aspirate the coated wells by inverting the plate and gently tap dry on tissue paper.
8. Block plate with 1× SuperBlock P20 Blocking Buffer (200 µL/well) for 1 h at room temperature.
9. Wash plate four times with 200 µL Washing Buffer per well, using a 200 µL multichannel pipette.
10. Thaw plasma samples on ice.
11. Centrifuge plasma 5 min at 10,000 g.
12. Dilute the supernatant of each plasma sample 1:1000* with 1× Assay Buffer.
13. Vortex to generate homogenous diluted plasma sample, briefly spin down to collect the volume on bottom of tube.

* It is recommended to perform an initial titration study to determine the optimal plasma dilution range for the specific experimental system. To do so, one approach would be to prepare a 5‐fold dilution series of plasma samples (for example: 1:50, 1:250, 1:1250, 1:6,250, 1:31,250, 1:156,250, 1:781,250 dilution etc., until background is reached) that are expected to contain the highest immunoglobulin levels (e.g., plasma collected after multiple EV injections).

14. Add 100 µL/well of diluted plasma samples.
15. Incubate plate at 37°C for 2 h.
16. Wash plate four times with Washing Buffer, 200 µL per well.
17. Dilute anti‐Monkey‐IgG‐HRP (1:10,000**) and/or anti‐Monkey‐IgM‐HRP (1:65,000**) with 1× Assay Buffer and add 100 µL/well to the washed plate.
18. Incubate at 37°C for 1 h.
19. Wash plate four times with Washing Buffer, 200 µL per well.
20. Add 100 µL/well TMB substrate.
21. Incubate plate for 6 min at room temperature, protect plate from light with aluminium foil.
22. Add 100 µL/well Stopping Solution.
23. Read absorbance of plate at 450 nm.

** Secondary antibody dilutions as recommended by manufacturer.

## 5 RESULTS

**FIGURE 1:**
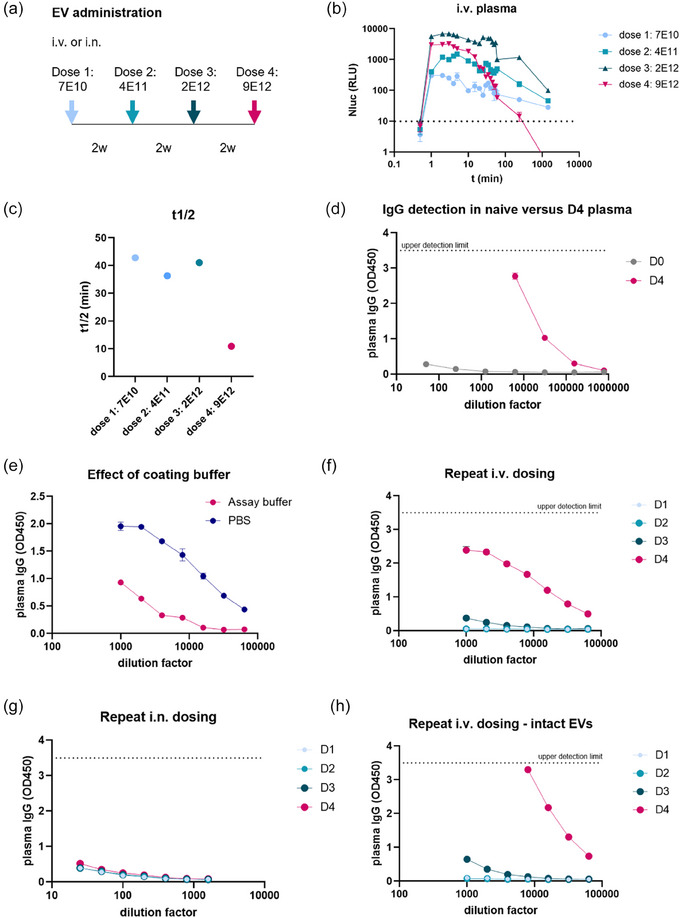
Repeated intravenous EV administration elicits an IgG response in macaque plasma (a) Four doses of EVs containing a palmBRET reporter protein were administered intravenously (i.v.) or intranasally (i.n.) into pig‐tailed macaques (*Macaca nemestrina*) at two‐week intervals, with increasing EV doses. In the 24 hours following each administration, blood plasma was drawn at regular intervals (Driedonks et al., 2022). (b) EVs were detected in plasma by measuring EV‐associated nanoluciferase signal in the timepoints after each EV administration. Graph was modified from (Driedonks et al., 2022). (c) EV half‐life was calculated from the data in B. Modified from (Driedonks et al., 2022). (d) Plasma obtained after the fourth i.v. administration (D4) and plasma of a naïve animal (D0) were serially diluted and used in the anti‐EV IgG ELISA described in this paper. (e) ELISA plates were coated with EV lysates that were diluted in commercial Assay Buffer or PBS. Serial dilutions of plasma from a macaque that received four i.v. EV doses were used to quantify anti‐EV IgGs. (f) Quantification of anti‐EV IgGs in plasma elicited after one, two, three, or four intravenous EV doses. (g) Quantification of anti‐EV IgGs in plasma elicited after one, two, three, or four intranasal EV doses. (h) ELISA plates were coated with intact EVs. Anti‐EV IgGs were quantified in plasma collected after one, two, three, or four intravenous EV doses.

Expi293F‐derived EVs containing a dual GFP/Nanoluciferase reporter (palmBRET) (Wu et al., 2020), were administered intravenously or intranasally to pigtailed macaques (*Macaca nemestrina*), as described previously (Driedonks et al., 2022). Plasma samples were collected at regular intervals in the 24 h after each administration. Four EVs doses were administered to each animal, with a two weeks interval between each dose (Figure 1a). We calculated the half‐life based on the EV‐associated nanoluciferase signal detected in the plasma samples collected in the 24 hours after each administration (Figure 1b). We noticed that the half‐life of the EVs in blood decreased after three administrations (Figure 1c), which motivated us to measure the levels of plasma immunoglobulins specific to EVs.

Since we observed reduced EV circulation times after three EV administrations, we used plasma that was collected after the fourth EV administration to set up the assay. Plasma from a naïve animal served as negative control. ELISA plates were coated overnight with EV lysates (equivalent of 1.8E8 EVs/well), and were blocked, after which serial dilutions of these plasma samples were added, followed by incubation with anti‐monkey‐IgG‐HRP (Figure 1d). Plasma collected after four intravenous administrations gave strong absorbance values between 50x and 31,250x dilution, and reached background levels above 156,250x dilution. Background absorbance was negligible in naïve plasma samples. This indicated the presence of IgGs specific to EV‐associated proteins in macaque plasma after four intravenous administrations.

The Assay Buffer contains a bovine protein that may interfere with efficient coating of the EV lysates on the ELISA plates. Therefore, we tested whether the sensitivity of the assay could be improved by diluting EV lysates in PBS instead of Assay Buffer (Figure 1e). Indeed, coating was more efficient in the presence of PBS than in Assay Buffer, so PBS was used in all subsequent experiments.

Next, we assessed whether the plasma IgG levels increased with repeated intravenous EV administrations. Plasma collected after each EV administration was serially diluted, and IgGs were quantified using our ELISA protocol (Figure 1f). After intravenous EV administration, anti‐EV IgGs could be detected at low levels after dose 3 and were strongly detected after dose 4. In contrast, after intranasal administration, anti‐EV IgGs could not be conclusively detected in plasma (Figure 1g). We then sought to determine if the IgGs were directed against one or more EV surface antigens, or against internal antigens. ELISA plates coated with intact EVs were used to detect IgG levels in the i.v. plasma samples (Figure 1h). We detected a strong IgG signal using intact EVs, indicating that the IgGs were directed against an epitope on the outside of the EVs. Together, these data suggest that repeated intravenous EV administration may lead to the generation of IgGs that recognize antigens on the outside of EVs.

## 6 DISCUSSION

We previously published the first study to indicate that repeated EV administrations may elicit antibody responses (Driedonks et al., 2022). This result may have been overlooked by previous studies for a variety of reasons. First, biodistribution studies in mice have generally used a single EV dose, and have mostly assessed tissue distribution within 24 h (Kang et al., 2021). Second, therapeutic studies regularly administer repeated doses (Table 1), but do not generally determine the clearance of EVs from the circulation. While the therapeutic effects that are reported in such studies (e.g., tumour regression) are generally encouraging, it would be interesting to know whether antibody responses are evoked, and whether preventing such immune responses would further increase the therapeutic efficiency of EVs. Third, cytokine responses and other readouts have generally been used to assess immune responses rather than detection of antibody responses.

**TABLE 1:** Non‐exhaustive list of studies performing more than three intravenous administrations of human cell‐derived EVs into non‐human animal models.

EV source	EV dose	Disease model	Animal species	# Admin.	Admin. intervals	Study runtime	Readout	Reference
Human K562	30 mg/kg	Melanoma	Mice	4	2 days	9 days	Inhibition of tumour growth, tissue necrosis	(Rivoltini et al., 2016)
Human umbilical cord MSC (PEG precipitation)	30 µg	Alzheimer's Disease	Mice	4	2 weeks	11 weeks	Spatial learning and memory, Aβ plaques in brain	(Ding et al., 2018)
Human 293T	100 µg	Colorectal cancer	Mice	5	3 days	30 days	Reduction in tumour volume	(Koh et al., 2017)
Human 293T	1E10 EVs	Encephalitis (EAE)	Mice	6	2 days	16 days	EAE paralysis score	(Gupta et al., 2021)
Human MSC	100 µg	Graft versus host disease (GVHD)	Mice	6	7 days	60 days	Survival rates, GVHD clinical score, histopathological damage	(Lai et al., 2018)
Human 293T (PEG precipitation)	200 µg	Liver fibrosis	Mice	8	4 days	4 weeks	Liver fibrosis, gene expression (miR‐155, TNF‐α, MCP‐1, and Col1α1)	(Li et al., 2020)
Human MSC	n.d.	Myocardial infarction	Pigs	14	12 h	7 days	Cardiac function	(Timmers et al., 2011)

It is not entirely clear whether the interval between administrations plays a role in the strength of IgG response. Our biodistribution study had two‐week intervals between i.v. administrations, which is comparable to vaccination regimens intended to generate strong antibody responses. In vaccination studies, longer intervals between priming and boosting have been associated with stronger immune responses (Brennan et al., 2012; Parry et al., 2022). Nevertheless, studies on PEGylated nanoparticles illustrate that shorter intervals may also elicit antibody responses. Weekly injection (7‐day interval) of PEGylated liposomes resulted in accelerated blood clearance (Dams et al., 2000). Furthermore, two injections of PEGylated EVs, 5 days apart, elicited IgM formation and consequently shorter circulation times (Emam et al., 2021). Studies of therapeutic EVs have used comparably short administration intervals (Table 1), making it plausible that anti‐EV IgGs have been formed. Our protocol will enable researchers to determine anti‐EV immune responses in their own experimental models.

We are convinced that our protocol will be of interest to the EV research community. The protocol does not require specialized equipment, and we envision that labs that test EVs in vivo will have ample surplus EVs to coat the ELISA plates. The protocol is easily adaptable to different experimental systems, for example, plasma from different animal species, EVs from non‐mammalian origins (e.g., bacterial EVs), and different biofluids (e.g., bronchoalveolar lavage). This protocol may help the community answer questions about how species differences between EV donor and EV recipient or cell source of EVs affect EV clearance and efficacy, and, if needed, to devise strategies to circumvent these potential roadblocks.

## CONFLICTS OF INTEREST

KWW is or has been an advisory board member of ShiftBio, Exopharm, NeuroDex, NovaDip, and ReNeuron; holds NeuroDex options; privately consults as Kenneth Witwer Consulting; and has a sponsored research agreement with Ionis Pharmaceuticals.
